# Comment on “Diagnosis of Sarcopenia by Evaluating Skeletal Muscle Mass by Adjusted Bioimpedance Analysis Validated With Dual‐Energy X‐Ray Absorptiometry” by Cheng et al.—The Authors Reply

**DOI:** 10.1002/jcsm.13865

**Published:** 2025-06-04

**Authors:** Simon Kwoon‐Ho Chow, Keith Yu‐Kin Cheng, Vivian Wing‐Yin Hung, Wing‐Hoi Cheung

**Affiliations:** ^1^ Department of Orthopaedics and Traumatology The Chinese University of Hong Kong Hong Kong SAR China; ^2^ Department of Orthopaeadic Surgery Stanford University Stanford California USA

We are writing in response to the comments by Kim [1] regarding our previous publication [2] in the *Journal of Cachexia, Sarcopenia and Muscle* (*JCSM*).

In our original publication, we reported our experience in screening sarcopenia patients according to the Asian Working Group on Sarcopenia (AWGS) 2019 definition [3]. During the course of the study, we realized that using the BIA in community settings without a DXA would result in a high rate of losing subjects due to false negatives. We have reached out to the manufacturer and tried to clarify the values and their interpretations, particularly the skeletal muscle mass (SMM) and the other segmental evaluations on lean muscle. However, responses were not assistive in providing a method to convert values shown in the report for screening purposes. The table below is a small, random subset of the subjects (*n* = 6) evaluated by the method provided in Kim's comments. According to Kim's clear explanations and instructions, ASM was calculated by the summation of the values for the appendages only in segmental lean analysis. ASM/height^2^ was then calculated to obtain the ASMI. This suggested method of obtaining the ASMI is still generating an average of 13.5% overestimation (calculation (F)) compared to the DXA values, resulting in 3 out of 6 false negatives according to the AWGS 2019 cut‐offs. In addition to this, we would like to point out a discrepancy between two parameters on the BIA InBody report with seemingly similar definitions, specifically the total lean mass in segmental lean analysis (by the summation of the mass of Right arm + Left arm + Trunk + Right Leg + Left leg) and the SMM which is the summation of the SMM of the appendages and the trunk according to Kim's suggested method. This discrepancy is found in both the example given by Kim as well as the samples, which we have summarized below. In Subject 1, for example, the SMM was 19.6 kg, while the total value in the lean segmental analysis was 28.23 kg. Since the ASM value utilized in Kim's method was the summation of the lean mass of the limbs in segmental lean analysis, it was implied that the total segmental lean analysis represents the SMM and should be the same as SMM. Clarifications as to whether segmental lean analysis includes other lean muscles or just the skeletal muscles could help with clearing up the different values and definitions.

**Table:** 

	Subjects		1	2	3	4	5	6	Average
	Age (years)		71	68	67	62	60	60	
	Sex (M, male; F, female)		F	F	F	M	F	F	
	Height (cm)		160	150	155	161	152	152	
From InBody Report	**BIA InBody 120**								
	SMM (kg)	From InBody report	19.6	16.1	19.9	23.6	18.5	17	
	Segmental lean analysis								
	L arm		1.21	1.00	1.71	2.21	1.5	1.3	
	R arm		1.4	1.11	1.8	2.21	1.61	1.3	
	Trunk		13.8	12.0	16.5	18.7	15.3	13.6	
	L leg		6.01	4.31	5.31	6.11	4.91	4.41	
	R leg		5.81	4.31	5.31	6.01	4.81	4.41	
	*Segmental lean total (kg)*	(Arms + legs + trunk)	28.23	22.73	30.63	35.24	28.13	25.02	
	**Calculations**								
(A)	*Total appendicular skeletal muscle mass (kg)*	(Arms + legs ONLY)	14.43	10.73	14.13	16.54	12.83	11.42	
(B)	*Ratio (total appendicular/SMM)*	(Total append. /SMM)	0.74	0.67	0.71	0.70	0.69	0.67	*0.7*
	**ASMI by BIA, DXA and BIA with prediction**								
(C)	*ASMI calculated by BIA (kg/m* ^*2*^ *)*	(Total append. /height^2^)	5.64	4.77	5.88	6.38	5.55	4.94	
(D)	*ASMI measured by DXA (kg/m* ^*2*^ *)*		4.97	4.21	4.23	6.07	4.64	4.93	
(E)	*Prediction model (ASMI, kg/m* ^*2*^ *)*	Model from publication	4.6	4.3	5	5.6	4.9	4.4	
	**ASMI difference compared to DXA**								
(F)	*% difference in ASMI (%) by BIA*	(BIA—DXA) /average	12.6	12.4	32.7	5.0	17.9	0.3	*13.5*
(G)	*% difference in ASMI (%) by prediction model*	(Prediction—DXA) /average	−3.9	1.1	8.3	−4.0	2.7	−5.7	*−0.2*

(A) = (total append. or ASM) the summation of L arm, R arm, L leg and R leg on the InBody BIA Report in the Segmental Lean Analysis, as instructed; (B) = The ratio of total append. (A) and SMM from the InBody BIA report; (C) = ASMI calculated by total appendicular skeletal muscle mass (A) divided by height in meter squared, as instructed; (D) = ASMI by DXA using segmental analysis including arms and legs only with cutlines defined by anatomical landmarks; (E) = ASMI calculated by prediction model in the publication; (F) = Difference in ASMI by BIA (calculated as instructed) compared to DXA; (G) = Difference in ASMI by BIA (calculated using the prediction model in the publication) compared to DXA.

Our group and many others are very supportive of using the BIA in community settings for fast, affordable, easy screening of sarcopenia for timely intervention where DXA is inaccessible. Therefore, we tried to compute and provide conversion equations to assist fellow healthcare providers a method to use the InBody systems directly for the estimation of ASMI, and for the diagnosis of sarcopenia. It has been widely recognized that all devices and instruments would have their limitations as demonstrated by the discrepancy in cut‐off values in ASMI using different evaluation methods. The current definitions as of the publication date may be the best of its time, however, are far from perfect. Therefore, the real problem we face is not the accuracy of the device, rather, the devices' ability to solve a clinical problem: If ASMI values can provide sufficient predictive power to detect a decline in physical function that is clinically significant. The authors would like to thank the JCSM for providing an open platform for this scientific discussion and an opportunity to raise concerns to improve our current practices.

Sincerely,

Simon K Chow et al.
